# Comparison of point-of-care thrombelastography versus conventional coagulation tests in the emergency department management of trauma

**DOI:** 10.1186/cc9858

**Published:** 2011-03-11

**Authors:** V Jeger, S Willi, T Liu, L Omert, A Orr, MA Popovsky, H Zimmermann, AK Exadaktylos

**Affiliations:** 1 University Hospital Inselspital Bern, Switzerland; 2 Haemonetics Corporation, Braintree, MA, USA

## Introduction

To guide the administration of blood products, coagulation screening of trauma patients should be fast and accurate. Conventional coagulation tests (CCT) are frequently not useful in the initial assessment of multiply injured patients, due to the delay in availability of results. The purpose of this study is to determine whether Rapid thrombelastography (RapidTEG^®^) results in 15 minutes correlate with Kaolin TEG or CCT.

## Methods

A 6-month prospective observational study of adult patients with suspected multiple injuries was conducted at a Level 1 trauma center of a university hospital. TEG, RapidTEG^®^, and CCT (INR, aPTT, TT, fibrinogen, platelet count) were performed within 10 minutes of the patient's arrival. Physicians blinded to TEG/RapidTEG^® ^results made the decision to transfuse based on clinical evaluation and prior threshold (cut-off) values for CCT. Cut-off values for RapidTEG^® ^were retrospectively assessed. Correlations between TEG and CCT and between TEG and RapidTEG^® ^parameters were calculated, as well as sensitivity and specificity of CCT and RapidTEG^® ^for any blood product transfused on day 1.

## Results

Seventy-six predominantly blunt trauma (96%, *n *= 73) patients comprised the dataset. The mean ISS was 18. Only weak correlation existed between CCT and relevant TEG parameters (*r *= 0.097 to 0.615). Strong correlation exists between Kaolin TEG and RapidTEG^® ^for K, MA, G and LY30 (*r *= 0.844 to 0.988). At the predetermined cut-off points for treatment in trauma, CCT demonstrated poor sensitivity. Cut-off points for RapidTEG^® ^demonstrated good sensitivity and specificity: RapidTEG^®^: Rapid K (seconds) 1.2; 80.0%; 59.2%; 0.785 (cut-off; sensitivity; specificity; AUC), Rapid angle (°) 74.7; 84.0%, 56.9%; 0.765, Rapid MA (mm) 61.5; 72.0%; 71.4%; 0.745. CCT: TT (seconds) 15; 28.6%; 88.9%; 0.529, aPTT (seconds) 60; 4.8%; 97.8%; 0.735, INR 1.5; 19.0%; 96.0%; 0.730.

## Conclusions

In this study of severely injured blunt trauma patients, RapidTEG^® ^can be utilized *in lieu *of kaolin TEG to provide faster test results. Cut-off points for treatment can be determined with RapidTEG^® ^to provide improved sensitivity and specificity compared with CCT with respect to blood product transfusion.

